# Prevalence, Risk Factors and Burnout Levels in Intensive Care Unit Nurses: A Systematic Review and Meta-Analysis

**DOI:** 10.3390/ijerph182111432

**Published:** 2021-10-30

**Authors:** Santiago Ramírez-Elvira, José L. Romero-Béjar, Nora Suleiman-Martos, José L. Gómez-Urquiza, Carolina Monsalve-Reyes, Guillermo A. Cañadas-De la Fuente, Luis Albendín-García

**Affiliations:** 1 Catalan Health Service, Vall d’Hebron Hospital, Passeig de la Vall d’Hebron, 119, 08035 Barcelona, Spain; santiago1992@correo.ugr.es; 2 Statistics and Operational Research Department, University of Granada, Avda. Fuentenueva S/N, 18071 Granada, Spain; 3 Nursing Department, Faculty of Health Sciences, Campus Universitario de Ceuta, University of Granada, C/Cortadura del Valle S/N, 51001 Ceuta, Spain; norasm@ugr.es (N.S.-M.); jlgurquiza@ugr.es (J.L.G.-U.); 4 Departamento de Ciencias Sociales, Universidad Católica de La Santísima Concepción, Avenida Alonso de Ribera, Concepción 2850, Chile; carolinamonsalve@ucsc.cl; 5 Nursing Department, Faculty of Health Sciences, University of Granada, Avda. de la Ilustración 60, 18016 Granada, Spain; gacf@ugr.es; 6 Andalusian Health Service, Granada-Northeast Health Management Area, Ctra. de Murcia S/N, 18800 Granada, Spain; lualbgar1979@ugr.es

**Keywords:** burnout, nurses, occupational health, risk factors, stress, intensive care unit

## Abstract

Nursing is considered to be an at-risk profession of burnout due to daily exposure to difficult situations such as death and pain care. In addition, some units such as the intensive care unit (ICU), can be stressful due to high levels of morbidity and mortality and ethical dilemmas. Burnout causes a deterioration in quality of care, increasing the risk of mortality in patients due to poor performance and errors in the healthcare environment. The aim of this study was to analyse the levels, prevalence and related factors of burnout in ICU nurses. A systematic review and meta-analysis were carried out in the Medline, Scopus and CINAHL databases. Fifteen articles were found for the systematic review and four for the meta-analysis. With a sample of *n* = 1986 nurses, the meta-analytic estimate prevalence for high emotional exhaustion was 31% (95% CI, 8–59%), for high depersonalization was 18% (95% CI, 8–30%), and for low personal accomplishment was 46% (95% CI, 20–74%). Within the dimensions of burnout, emotional exhaustion had a significant relationship with depression and personality factors. Both sociodemographic factors (being younger, single marital status, and having less professional experience in ICU) and working conditions (workload and working longer hours) influence the risk of burnout syndrome.

## 1. Introduction

In 1974, the American psychiatrist Freudenberger referred to burnout as a state of fatigue or frustration that appears after dedication to a cause, lifestyle, or relationship in which the expected effort is not produced [1]. Burnout appears as a negative response caused after chronic work episodes of stress. Professionals with this syndrome perceive it as a constant imbalance between their needs, their values and the work they do [2].

This phenomenon has three main components: emotional exhaustion (EE) or gradual loss of energy, exhaustion, tiredness and fatigue, which is externalized in a physical or psychological way, or both; depersonalization (D) or negative behaviours along with indifferent responses towards those who receive care or treatment; and low personal accomplishment (PA) that consists of being limited and not feeling fulfilled with the work performed [2]. There are also other burnout measurement tools such as Copenhagen Burnout Inventory [3].

In 2001, Maslach et al. [4] developed a validated instrument, the Maslach Burnout Inventory (MBI), to measure burnout syndrome. It consists of a questionnaire that evaluates the three components described (EE, D, and PA) through 22 items using a 7-point Likert-type scale (where 0 is never and 6 is every day). A high score in EE and D, and a low level in PA indicate the presence of burnout, and it can be classified into different levels as it is an ordinal variable: low, medium and high [4].

Burnout is related to physical disorders such as respiratory, heart, and intestinal pathology, headaches, type 2 diabetes, hypercholesterolemia, prolonged fatigue, and muscle pain [5]. Burnout has also a negative effect on mental health causing depressive and anxiety symptoms, alcohol consumption, insomnia, and even suicidal ideas [6,7]. In addition, burnout syndrome can cause absenteeism and job dissatisfaction [8,9].

Nursing is considered to be a risk profession due to daily exposure to difficult situations such as death and pain care [10]. In particular, intensive care units (ICU) can be stressful due to high levels of mortality, critical medical condition, and ethical dilemmas [11]. This situation can increase when the staff does not require enough time to provide adequate care for each patient [12].

The chronicity of burnout syndrome can lead to provoke deep emotional reactions, that without intervention or guidance aimed at its prevention, can cause damage to the physical and psychological integrity of the health professional [10]. Burnout can also tend to cause a deterioration in the quality of care, increasing the risk of morbidity and mortality in patients, due to poor performance and increased errors in the healthcare environment [13]. In this sense, it is interesting to carry out a review about burnout in ICUs. This syndrome has been classified as an occupational phenomenon; thus, its prevention through the implementation of coping strategies is essential [12].

The objective of this study was to analyse the levels, prevalence, and related factors of burnout in intensive care nurses.

## 2. Materials and Methods

### 2.1. Design and Search Strategy

A systematic review with meta-analysis was performed following the PRISMA recommendations (Preferred Reporting Items for Systematic Reviews and Meta-analyses) [14]. The search was carried out through the databases Medline (PubMed), Scopus (Elsevier), and CINAHL (EBSCO). Using the MeSH terms the search strategy was “burnout AND nurses AND intensive care units”. The search was conducted in December 2020.

### 2.2. Inclusion and Exclusion Criteria

The inclusion criteria were: (1) primary quantitative studies that measured burnout and its risk factors; (2) sample of nurses of intensive care unit nurses; (3) data of level; prevalence; or related factors; (4) published in English and Spanish language; (5) published in the last five years; (6) studies using the Maslach Burnout Inventory (MBI) were included in the meta-analysis. The exclusion criteria were: (1) systematic or bibliographic reviews; (2) studies carried out in paediatric or neonatal intensive care units; (3) studies that included different health professions without specific data for nurses.

### 2.3. Study Selection, Quality Appraisal, and Risk of Bias

For the selection of the studies, first, a screening was carried out based on the duplicate studies. In the article selection process, two of the authors (S.R.-E. and N.S.-M) independently reviewed the title and abstract of the articles found. Finally, the full text was read. A third author (J.L.R.-B) was consulted in case of disagreement.

The quality of the articles selected for this review was evaluated using the levels of evidence and grades of recommendation stipulated by the Oxford Centre for Evidence- Based Medicine (OCEBM) [15]. Table 1.

### 2.4. Data Abstraction

Two authors (S.R.-E. and N.S.-M.) collected data from each selected study. A third author verified the data in case of disagreement (L.-A.-G.). The following collected as data were obtained for each included article: year of publication, country of study, type of study, sample size, and main results.

For the systematic review, a descriptive analysis of the data included in the studies was performed. StatsDirect software for Windows was used for the meta-analysis. A random effects meta-analysis of the prevalence of high EE, high D, and low PA was performed with the data from the total study sample and the prevalence of burnout syndrome. Heterogeneity was analysed with the I2 test, publication bias with the Egger test, and a sensitivity analysis was performed.

## 3. Results

In total, 237 articles were found. After screening by title and abstract and reading the full text, a total of 15 studies were included for the systematic review.

**Figure 1:**
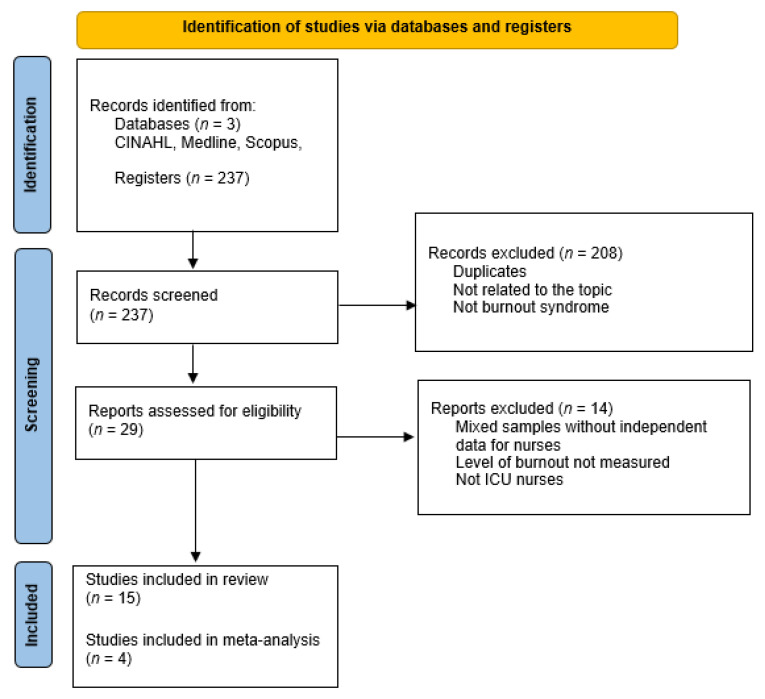
Flow diagram of the publication search process.

The selection process of included studies is shown in Figure 1. Most of the studies selected for this review were conducted in Brazil (*n* = 4), Spain (*n* = 3), and South Korea (*n* = 2), with the remainder conducted in Turkey, China, Greece, Chili, Italy, and India. The detailed information of each study is shown in Table 1.

### 3.1. Prevalence of Burnout Syndrome

In the articles selected, there were several instruments to measure burnout syndrome. Among them, there was high use of MBI in 87.5% of studies. The prevalence of burnout varies depending on the country where the research was carried out. Two studies carried out in Brazil showed disparate results, in one low level of burnout, in all its dimensions [25], and in the other, a high prevalence of burnout [19]. In another study carried out in India, most of the participants obtained a high level in the three dimensions established by the Maslach questionnaire [28]. Burnout levels of each article are shown in Table 1.

### 3.2. Socio-Demographic Factors

Sociodemographic factors such as being younger, single marital status, and having less professional experience in the intensive care unit (ICU were associated with burnout levels [24,25,27,29]. Age was negatively correlated with EE, and the effort–reward imbalance was positively correlated with EE [27]. For nursing professionals, for each year of work that they spent in the ICU, there was an increase in the levels of low PA [21]. Other related factors of burnout were a lack of time for physical exercise, work in a high-quality hospital, the presence of comorbidities, and years of experience [18].

### 3.3. Personality Related Factors

A high EE score was significantly related to depression and personality factors [18,29,30]. Some authors showed that EE was significantly related to four personality factors, neuroticism, agreeableness, responsibility, and extraversion. Likewise, the D and PA were related to these same factors by adding openness [18].

Neuroticism was a positive predictor of EE [18,26] and extraversion was a negative predictor [26]. Conversely, EE was interrelated with depression, job stress, and job satisfaction within intensive care nursing staff [29]. Depressive symptoms are more likely to appear in nurses who work in the ICU and who have a high level of burnout [26,30].

### 3.4. Occupational Related Factors

Regarding working hours among nurses who perform complementary physical days, 15.3% presented high levels of EE, 17.9% high D, and 65.9% low PA. The percentages of those who did not perform complementary physical work were 10.8%, 11.8%, and 61%, respectively. These data reflect that high work overload was related to high levels of EE and D [17]. In the same way, high EE and low PA were associated with an increase in the workload, reflected in a greater number of patients [21].

### 3.5. Other Outcomes of Interest

Some authors analysed resilience as a way of adapting to adverse situations. Resilience was negatively related to D and positively to PA. A higher level of resilience was related to an improvement in nurse mental health, as well as the willingness to work effectively [16].

Other studies also studied the relation of burnout with the infection rate in ICU [22], or the prevalence between ventricular and atrial extra systoles in ICU nurses [20], although the results showed no relationship.

### 3.6. Meta-Analysis of the Prevalence of High EE, High D and Low PA

Four studies included the information necessary for the meta-analysis, with a total sample of *n* = 1986 nurses. Egger’s test showed no publication bias, and no studies were excluded after sensitivity analysis. Heterogeneity in the three meta-analyses performed was high, therefore a random-effects meta-analysis was used.

**Figure 2:**
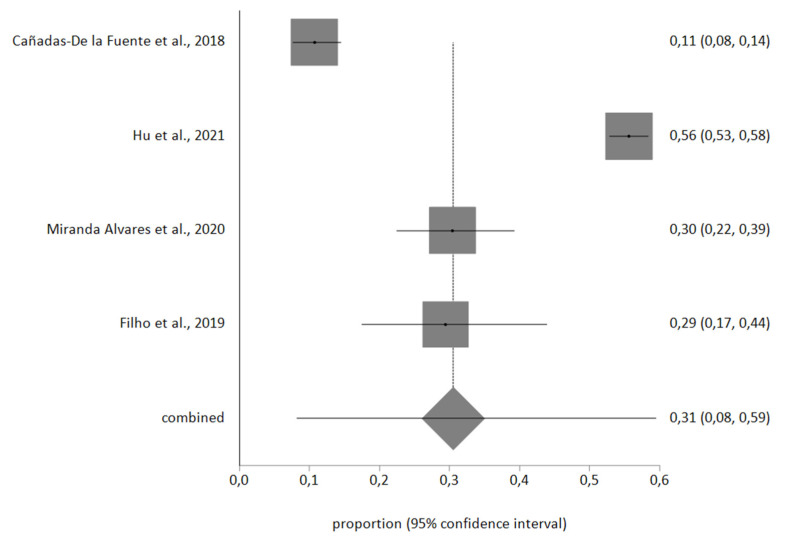
High *EE* forest plot.

**Figure 3:**
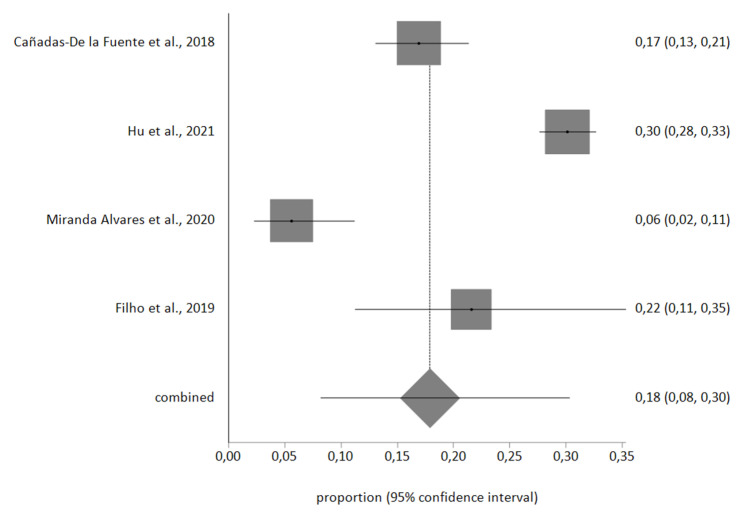
High *D* forest plot.

**Figure 4:**
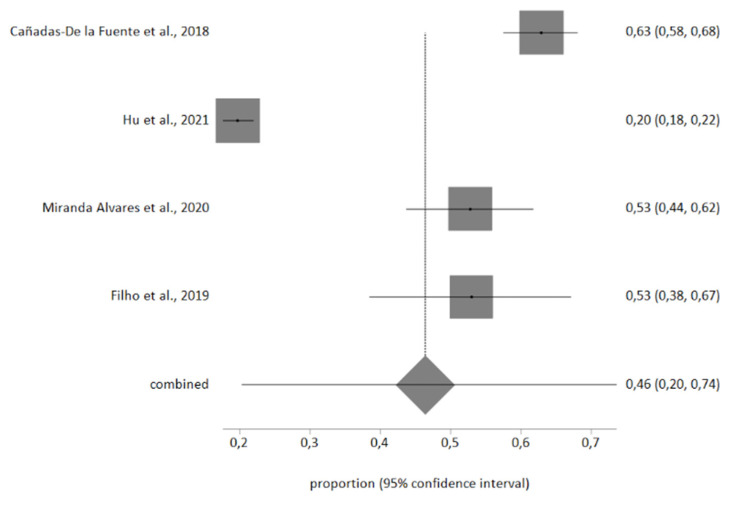
Low *PA* forest plot.

The meta-analytic estimate prevalence for high EE was 31% (95% CI, 8–59%), for high D was 18% (95% CI, 8–30%), and finally for low PA was 46% (95% CI, 20–74%). The forest plots are shown in Figure 2, Figure 3 and Figure 4.

## 4. Discussion

The prevalence of burnout in the included studies differs between countries and health systems where the studies were performed. Similarly, in other reviews carried out in other nursing areas, there were similar differences [31,32,33], although, these data are inferior to nurses from other services, such as mental health, paediatrics, oncology or primary care [34,35,36,37]. Given that the health system of each country has different characteristics, competencies in the nursing area, training programs, workload, and costs of care, the levels of burnout can be diverse [38,39].

As demographic factors, we found work overload, seniority, and age as main factors related to burnout syndrome in ICU nurses. As corroborated by other studies, the excessive number of daily working hours and a high workload, in addition to a lack of time for patient care, produced emotional disorders and low job satisfaction [40,41]. In critical care service, the emotional and physical domains are overloaded due to the complex labour interventions carried out by nursing staff [42,43]. Other authors highlight that high levels of EE were related to personal factors such as being single and caring for children, as well as work factors such as long working days, poor quality work life, and a lack of time to dedicate to self-care [44]. In relation to the experience, some studies indicated that older professionals with a permanent job showed a high EE related to the daily routine within the workplace, stress, and high workload [45]. An adequate work environment, with good working relationships [46] and support by the institution [47], were found as protective factors. Other authors also found that an improvement in salary [48] leads to improved motivation [49]; as a consequence, job satisfaction increases, and professionals are less prone to suffer burnout syndrome [50].

In relation to the psychological factors, ICU nurses with high levels of anxiety and depression have shown high levels of burnout. Work practice is faced with an exhausting and unfavourable work environment that deteriorates the professional’s quality of life, adding a lack of interest and frustration that provokes leaving the job [51]. Thus, it is essential to provide interventions to improve mental health and promote coping strategies [52,53].

The study had some limitations. The number of articles included in the meta-analysis was low. The heterogeneity (I2 index) was high because population samples of included studies were analysed in different countries with diverse healthcare systems. The results also reflected the working conditions of each geographical area, an important aspect for its subsequent interpretation. Another limitation was that not all studies used the same measurement questionnaire; therefore, it was not possible to combine all the results, the meta-analytic estimate of the prevalence measured only with MBI was analysed.

## 5. Conclusions

ICU nurses tend to have high emotional exhaustion and low personal accomplishment. The main sociodemographic factors related to burnout syndrome were being younger, single marital status, and having less professional experience in the ICU, and the work-related factors were workload and working longer hours. Emotional exhaustion had a significant relationship with depression and personality factors. The work environment had a great influence on reducing the prevalence of burnout; therefore, support and job satisfaction, as well as an improvement in the worker’s self-esteem, can be protective factors. Health policy should develop interventions and training to improve working conditions, the environment, and coping skills in order to improve the quality of patients and professionals care.

## Figures and Tables

**Table 1:** Characteristics of the included studies (*n* = 15).

Author, Year, (Country)	Study Design	Sample	Burnout Prevalence	Main Results	OCEBM * LE/GR	
Arrogante & Aparicio, 2017 (Spain) [16]	Cross-sectional study	*n* = 30 ICU nurses	Levels of emotional exhaustion and depersonalization were medium and personal accomplishment level was high	Emotional exhaustion and depersonalization were negatively related to mental health, resiliency, and physical health Personal accomplishment was positively related to mental health and resiliency	2c/B	
Cañadas-De la Fuente et al., 2016 (Spain) [17]	Cross-sectional study	*n* = 1225 ICU nurses	44.1% were in the highest levels of the syndrome	Nurses with extra shifts were more prone to high levels of emotional exhaustion, depersonalization, and low personal accomplishment	2c/B	
Cañadas-de la Fuente et al., 2018 (Spain) [18]	Cross-sectional study	*n* = 337 ICU nurses	High Emotional Exhaustion 11% High Depersonalization 17% Low personal accomplishment 63.3%	Personality factors and depression were related with high levels of burnout Emotional exhaustion was statistically related to neuroticism, amiability, responsibility, extraversion and with anxiety and depression Depersonalization and personal accomplishment were related to the five personality factors and with anxiety and depression High Burnout level was associated with neuroticism and amiability	2c/B	
Da Silva et al., 2017 (Brazil) [19]	Cross-sectional study	*n* = 130 ICU nurses	Burnout prevalence 55.3%	-	2c/B	
Denat et al., 2016 (Turkey) [20]	Cross-sectional study	*n* = 51 ICU nurses	Nurses showed low levels of emotional exhaustion, low depersonalization, and medium personal accomplishment	There was no relationship between burnout and ventricular extrasystoles or atrial extrasystoles	2c/B	
Filho et al., 2019 (Brazil) [21]	Cross-sectional study	*n* = 209 ICU nurses	High emotional exhaustion 29.4% High depersonalization 22% Low personal accomplishment 52.9%	Each patient added to nurses workload was associated with a 67% and 37% increase in the probability of emotional exhaustion and low personal accomplishment Each year working at the ICU was associated with a 7% higher probability of low personal accomplishment	2c/B	
Galleta et al., 2016 (Italy) [22]	Cross-sectional study	*n* = 101 ICU nurses	53% emotional exhaustion and 61.5% medium or high levels of depersonalization	Burnout was related with psychosocial aspects and with hospital rate infection	2c/B	
Hu et al., 2021 (China) [23]	Cross-sectional study	*n* = 1315 ICU nurses	High Emotional Exhaustion 56.6% High Depersonalization 30% Low Personal accomplishment 20%	Professionals working in ICU were more prone to burnout. The associated factors were low frequency of physical exercise, having comorbidities, working in a high-quality hospital, having more years of professional experience, night shifts, and having fewer holidays	2c/B	
Kim & Yeom, 2018 (South Korea) [24]	Cross-sectional study	*n* = 318 ICU nurses	Burnout levels was 3.15 in a scale to 5	Higher burnout levels were significantly associated with being younger, unmarried, having a lower education level, lower professional experience, and having no religion Higher levels of spiritual well-being were related with lower burnout	2c/B	
Miranda Alvares et al., 2020 (Brazil) [25]	Cross-sectional study	*n* = 125 ICU nurses	High Emotional Exhaustion 30% High Depersonalization 6% Low personal accomplishment 53% Burnout prevalence was 0.41% following Maslach criteria	Being 35 years old or more showed a lower probability of emotional exhaustion and depersonalization More working hours were related to lower personal accomplishment Men had lower personal accomplishment Less physical activity was related to higher emotional exhaustion and depersonalization	2c/B	
Ntantana et al., 2017 (Greece) [26]	Cross-sectional study	*n* = 493 ICU nurses	Burnout was present in 2.6% Exhaustion was higher in nurses than physicians	Neuroticism was positively related to exhaustion and extraversion was negatively related to exhaustion. Personality factors, labour satisfaction, and end-of-life care are related to burnout	2c/B	
Padilla Fortunatti & Palmeiro Silva 2017 (Chile) [27]	Cross-sectional study	*n* = 36 ICU nurses.	-	Emotional exhaustion was negatively related to age and positively related to an imbalance between effort-reward; depersonalization was negatively related to age	2c/B	
Saravanabavan et al., 2019 (India) [28]	Cross-sectional study	*n*= 164 ICU nurses	69% nurses had burnout	Laboral satisfaction was significantly correlated with Burnout; stress levels were correlated with emotional exhaustion and depersonalization	2c/B	
Sok et al., 2020 (South Korea) [29]	Cross-sectional study	*n* =115 ICU nurses	The mean score of burnout was 64.03 in the Copenhagen Burnout Inventory, indicating a high level	Burnout had a significant and positive relationship with depression and job stress and a negative significant relation with age	2c/B	
Vasconcelos et al., 2018 (Brazil) [30]	Cross-sectional study	*n* = 91 ICU nurses	14.3% showed burnout	Higher levels of depression were correlated with higher levels of emotional exhaustion and depersonalization and with lower levels of personal accomplishment	2c/B	

*—levels of evidence of the Oxford Centre for Evidence-Based Medicine; LE, level of evidence; GR, grade of recommendation.
